# Editor's choice: Covid-19 and HIV are still very much with us

**DOI:** 10.4314/ahs.v23i2.1

**Published:** 2023-06

**Authors:** James K Tumwine

Infections continue plaguing our continent and overwhelming the fragile health systems. That is why we continue publishing large numbers of papers on this subject. Clearly there is need to have a re-look at this, since non communicable diseases also have reached silent and overt epidemic levels. It is with is in mind that we have a very interesting menu of papers in this June 2023 issue of African Health Sciences. Thus COVID-19 contributes six papers^1^–^6^, as has HIV^7^–^12^. Tuberculosis^1^–^15^, on the other hand, remains an important infection as are hepatitis^16^–^17^, Human papilloma virus^18^, pneumococcal disease^19^ and others^20^–^26^.

Putting infections aside we have other interesting papers. First cancer^27^–^35^. The cancer papers touch on cervical cancer^27^, ovarian tumors^28^, hematological malignancy; others^29^–^35^; and surgery^36^–^49^. These are followed by ocular conditions^50^–^54^. The next group has diabetes mellitus^55^–^58^; and cardiovascular disease^59^–^62^. Obesity and physical inactivity^63^–^64^ come in light of recent allegations that the science inherent in body mass index (BMI) is racist.

The next NCDs are on Parkinson's disease^65^, NCD knowledge^66^ and others^67^-^69^. The next section deals with sexual and reproductive health issues: late antenatal booking in Tanzania^70^, pregnancy outcome of in vitro fertilization^71^; caesarian section births^72^-^73^.

Assisted reproductive health services^74^ and men's health issues^75^ conclude this section. The next section reflects child health issues^76^-^82^. The treatise continues with teaching and artificial intelligence^83^-^85^; and job satisfaction among rehabilitation staff^86^. Finally we appeal to our stakeholders to be patient as we strive to clear the backlog of papers that accumulated during the Covid-19 pandemic.
